# Real-time mechanical characterization of DNA degradation under therapeutic X-rays and its theoretical modeling

**DOI:** 10.1038/micronano.2016.62

**Published:** 2016-12-05

**Authors:** Grégoire Perret, Thomas Lacornerie, Fabio Manca, Stefano Giordano, Momoko Kumemura, Nicolas Lafitte, Laurent Jalabert, Mehmet C. Tarhan, Eric F. Lartigau, Fabrizio Cleri, Hiroyuki Fujita, Dominique Collard

**Affiliations:** 1 LIMMS/CNRS-IIS UMI 2820, Institute of Industrial Science, The University of Tokyo, 4-6-1 Komaba Meguro Ku, Tokyo 153-8505, Japan; 2 IEMN, UMR8520, CNRS, Avenue Poincaré Cité Scientifique, BP 60069, Villeneuve d’Ascq, Cedex 59652, France; 3 CNRS/IIS/COL/Lille 1 SMMiL-E project, CNRS Délégation Nord-Pas de Calais et Picardie, 2 rue de Canonniers, Lille, Cedex 59046, France; 4 Centre Oscar Lambret, Université de Lille, Département Universitaire de Radiothérapie, Centre Oscar Lambret, Lille 59000, France; 5 Institute of Industrial Science, The University of Tokyo, 4-6-1 Komaba, Meguro-ku, Tokyo 153-8505, Japan

**Keywords:** biomechanical measurements, DNA damage, microfluidic, radiotherapy, real-time, Silicon Nanotweezers

## Abstract

The killing of tumor cells by ionizing radiation beams in cancer radiotherapy is currently based on a rather empirical understanding of the basic mechanisms and effectiveness of DNA damage by radiation. By contrast, the mechanical behaviour of DNA encompassing sequence sensitivity and elastic transitions to plastic responses is much better understood. A novel approach is proposed here based on a micromechanical Silicon Nanotweezers device. This instrument allows the detailed biomechanical characterization of a DNA bundle exposed to an ionizing radiation beam delivered here by a therapeutic linear particle accelerator (LINAC). The micromechanical device endures the harsh environment of radiation beams and still retains molecular-level detection accuracy. In this study, the first real-time observation of DNA damage by ionizing radiation is demonstrated. The DNA bundle degradation is detected by the micromechanical device as a reduction of the bundle stiffness, and a theoretical model provides an interpretation of the results. These first real-time observations pave the way for both fundamental and clinical studies of DNA degradation mechanisms under ionizing radiation for improved tumor treatment.

## Introduction

In the early days of radiotherapy, very little was known about the mechanism of action mechanism of ionizing radiation and its side effects, sometimes leading to disastrous results. The understanding of molecular genetics made it clear that radiation mainly alters the DNA of cells, mostly causing oxidative damage^1,2^. However, as radiation does not distinguish between healthy and tumor cells, the largest improvement in radiotherapy technology in modern days has concentrated on improving the precision of dose-delivery in space and time, with considerably less interest in the fundamental understanding of the basic mechanisms of biological radiation damage. We need a deeper understanding of the direct molecular-scale correlations between the radiation dose and biological damage to open the way to more efficient and customized patient-specific radiation treatments.

Among the main experimental tools adopted up until now to study radiation damage in DNA at the molecular level, we find gas chromatography with ion-selective mass spectrometry^3^, high-performance liquid chromatography^4^, and electron paramagnetic spin resonance^5,6^. All such methods are based on chemical treatments performed on DNA after irradiation. Typically, DNA samples are irradiated by a known dose, stored for long times, and subsequently hydrolyzed and derivatized. The resulting solution contains the individual DNA bases, both damaged and undamaged, to be analyzed by one of the above methods. The link between radiation damage and the molecular structure of DNA, however, is very indirect (chemical signatures correlated to the damage are observed) and subject to the variable conditions of subtle shifts in the oxidation paths (differential recombination^7^).

Our working hypothesis is that radiation damage should induce specific signatures that can be detected by changes in the micromechanical response of the molecules. On one hand, direct mechanical characterization of DNA is now routinely performed by biophysical instruments, such as an atomic force microscope^8^, optical tweezers^9^, or magnetic tweezers^10^, which are extremely accurate and can achieve single-molecule level resolution. On the other hand, these instruments are practically unusable for measuring radiation–DNA interactions since they operate on individual molecules, although radiation damage is a statistical event. Moreover, these instruments are bulky, rather expensive, and cannot operate in confined areas and severe conditions. To fulfil the needs of molecular manipulation and biomechanical measurement in the harsh environment of a radiation beam, low-cost and large-scale manufactured micro-electro mechanical systems (MEMS) may be a more appropriate approach^11,12^. In this study, we introduce and fully characterize a MEMS-based device, the Silicon Nanotweezers (SNT)^13^, as an ideal instrument to perform the unprecedented real-time biomechanical detection of the radiation damage of DNA exposed to the ionizing radiation environment of radiotherapy treatment. In short, we will employ an integrated MEMS device, the SNT^13,14^, to directly measure the break rates of DNA placed under an ionizing radiation beam. DNA bundles of a known sequence, with lengths in the micron range, will be trapped and held straight in parallel strands between the tips of the SNT device. Such nanoscale vibrating tips can measure the elastic modulus of the DNA bundle immersed in physiological water in a microfluidic cavity^15,16^. Under well-known and fully characterized irradiation conditions provided by clinical radiotherapy machines, individual DNA strands in the bundle will accumulate damage and break, thus progressively reducing the bundle mechanical strength. Correspondingly, the SNT device will measure the dynamic mechanical response of the bundle in real-time, with a time-constant characteristic of the different damage types. Moreover, the experimental results will be interpreted with a theoretical model of a randomly damaged DNA bundle to assess the relevance of the real-time biomechanical characterizations^17^.

## Materials and methods

The novel approach of this study relies on the real-time biomechanical sensing capability of the SNT that is integrated in an experimental setup installed in the hospital environment under a radiotherapy linear particle accelerator (LINAC) machine. The portable characteristics due to its tiny size of 35 mm^2^ and peripheral electronics equipment make the SNT an excellent candidate for in-beam operation.

### The Silicon Nanotweezers

The SNT, a MEMS device for the direct manipulation of biomolecules, consists of three important parts for (i) biomolecule handling, (ii) mechanical actuation, and (iii) displacement sensing. To avoid a damping effect in liquid, the immersion has to be restricted. The sharp protruding tip geometry is preferred to minimize the immersion of the tips of the SNT arms.

Standard micromachining techniques are performed to fabricate the SNT as described elsewhere^18^. Using a <100> oriented silicon-on-insulator (SOI) wafer, the movable structures are fabricated by deep reactive ion etching (DRIE) and vapour hydrofluoric acid releasing. Sharp tips require local oxidation of silicon and wet anisotropic etching.

**Figure 1:**
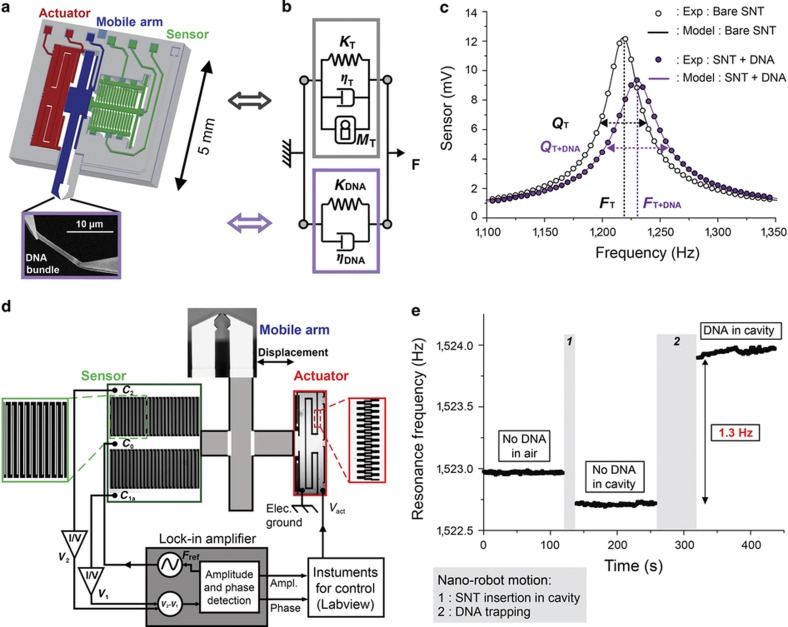
Silicon Nanotweezers (SNT) and DNA mechanical characterization in liquid. (**a**) Schematic view of the main parts of the SNT. The displacement is provided by comb-drive actuators and measured by a differential capacitive sensor. Opposing tips are used for handling biomolecules, for example, DNA molecules as shown with a scanning electron microscope image. (**b**) Damped oscillator models of the SNT (in gray) and DNA (in purple). (**c**) Frequency response of the bare SNT and SNT with DNA. The model provides the quality factor (*Q*) and resonance frequency (*F*) from the frequency response to calculate the mechanical properties of the DNA bundle. (**d**) Schematic view of the electrical set-up. The outputs of differential capacitive sensors are fed into the lock-in-amplifier to drive the actuator using LabVIEW software. (**e**) Real-time resonance frequency monitoring. Starting from bare SNT measurements in air, the nano-robot moved (1) to insert the SNT into liquid. After trapping a DNA bundle (2), the measurements continued in liquid.

The gap between the opposing tips (8–15 μm) is designed to match the target biomolecules, that is, λ-phage DNA (with a length of 16 μm; Figure 1a). One arm is mobile (highlighted in blue) and displaced by an electrostatic comb-drive actuator (in red). The motion of the arm is acquired by a displacement sensor^17^ (in green). The SNT is modelled as a dumped oscillator (gray-circled block in Figure 1b). Its main parameters are the mass of the mobile part (*M*_T_), total viscosity (*η*_T_) due to friction and air damping, and stiffness (*K*_T_=25 N m^−1^) of the mobile arm (comparable to the stiffness of a DNA bundle). The load represented by the trapped DNA molecules between the SNT tips is modelled with additional stiffness (*K*_DNA_) and viscosity (*η*_DNA_) in parallel (purple-circled block in Figure 1b) on the SNT model. The frequency response (Figure 1c) shows a main resonance frequency, *F*_T+DNA_, of the system with a quality factor, *Q*_T+DNA_. These parameters are directly related to the mechanical characteristics of the SNT and the molecules eventually trapped between its tips. As the frequency response of the bare SNT is known (*F*_T_ and *Q*_T_), the molecular bundle mechanical characteristics (stiffness and viscosity) can be extracted using the well-established damped oscillator model^19^. When the tips of the SNT (without DNA) are inserted in the solution (Figure 1e), the resonance frequency of the bare SNT drops owing to the added mass^20^. This resonance frequency shift also encompasses the meniscus effect. This SNT response is stable and acts as the reference for the extraction of the bundle characteristics in solution.

A lock-in-amplifier performing a phase-lock loop (Figure 1d) receives the signal from the capacitive displacement sensor and drives the actuator to monitor the resonance frequency (*F*_T+DNA_) and quality factor (*Q*_T+DNA_) of the entire SNT+DNA system in real-time. Thus, the mechanical characteristics of the molecules (stiffness and viscosity) can be extracted in real-time by using the following equations:

(1)KDNA=KTFT+DNA2−FT2FT2

(2)ηDNA=KT+KDNA2π.QT+DNA.FT+DNA−ηT

**Figure 2:**
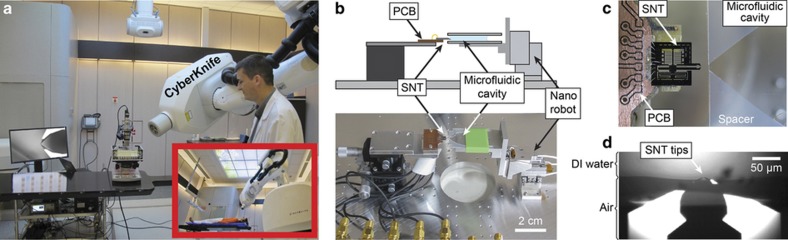
SNT and microfluidic set-up in the hospital. (**a**) Set-up on a patient bed support. The medical physicist focuses the beam direction of the CyberKnife on the tips of the SNT. (**b**) The SNT is aligned in front of the microfluidic cavity. (**c**) The top view of the SNT aligned to insert the tips into the cavity. (**d**) Only the tips of the SNT enter the liquid so that the actuators and sensors can provide their in-air performance.

As the CyberKnife LINAC machine is dedicated to therapeutics, its use for research purposes is possible in extremely limited time periods. Therefore, a time-efficient experimental process is critical. To optimize the protocol, the set-up is pre-installed and automated to take advantage of free session times of the CyberKnife LINAC provided by the medical physicist (Figure 2a). Each experiment starts with the calibration of the bare SNT; its *F*_T_ and *Q*_T_ characteristic values are recorded both in air and deionized water. Then, a DNA bundle is trapped and inserted inside the microfluidic cavity (as detailed below). The observed frequency shift (1.3 Hz for the case shown in Figure 1e) represents the practical range for the observation of DNA degradation. The SNT is subsequently placed under the CyberKnife head (Figure 2a). The collimated beam completely encompasses the SNT holding the DNA bundle. Different irradiation sequences have been tested with this configuration. Although both stiffness and viscosity values are obtained, only the stiffness is reported in this paper.

### The CyberKnife

The Department of Radiation Therapy of Centre Oscar Lambret provided a CyberKnife for the DNA irradiation. The 6 MV LINAC head, attached on a robotic arm, uses circular collimators between 5 and 60 mm. The dose rate is 8 Gy min^−1^ at a distance of *d*=80 cm from the X-ray source for the largest collimator. The dose rate varies with the collimator opening and follows the *d*^−2^ divergence law of the free propagation of the beam. For each irradiation cycle, the dose is calculated from the SNT position and calibrated collimation effect. The small size of the 5 mm collimator has the strong advantage of minimizing the noise due to irradiation on the SNT. For the same purpose, the electronic equipment is placed 1 m away from the beam. Finally, the three-dimensional (3D) robot of the CyberKnife simply positions the set-up under the beam.

### The microfluidic cavity

During an irradiation session, a strong predominance of indirect damage (by free radicals originating from water radiolysis), as opposed to the direct radiation damage of DNA strands, has been demonstrated^2^. The most lethal damage, leading to mechanical breaking of the DNA molecule, is the double-strand break (DSB), which can in fact be induced by both direct and indirect effects. To generate both types of DSB damage during our experiments, the DNA must be placed in hydration conditions. The insertion of the DNA inside solution provides appropriate hydration conditions, which approximate those of the cytoplasm. To facilitate the stable immersion of DNA in the solution, a microfluidic cavity is designed (Figures 2b and c) to introduce the SNT inside the liquid before DNA irradiation.

The microfluidic cavity is assembled using two cover slips with two pieces of silicone rubber (0.5 mm height) as spacers forming a reservoir. The assembled microfluidic cavity has a small opening (1 mm×0.5 mm) in the front and a large opening (~20 mm×0.5 mm) in the back. The SNT tips are inserted into the liquid through the small opening. This small opening pins the meniscus enabling a stable position of the tip/solution interface and a constant *F*_T_ reference during the experiment. The evaporation mainly occurs on the larger opening (20 mm) without any noticeable effect on the stability of the measurements within the experimental period. In addition, a small slot (5 mm×0.5 mm) on the corner of the front side is used for the DNA trapping protocol.

The SNT must be inserted inside the microfluidic cavity at an optimal and constant position in each experiment, and it must be precisely maintained for the entire duration of the measurement to compare different experimental runs. A 3D positioning robot based on piezoelectric elements provides nanometre accuracy and a semi-automated manipulation.

### DNA trapping protocol

**Figure 3:**
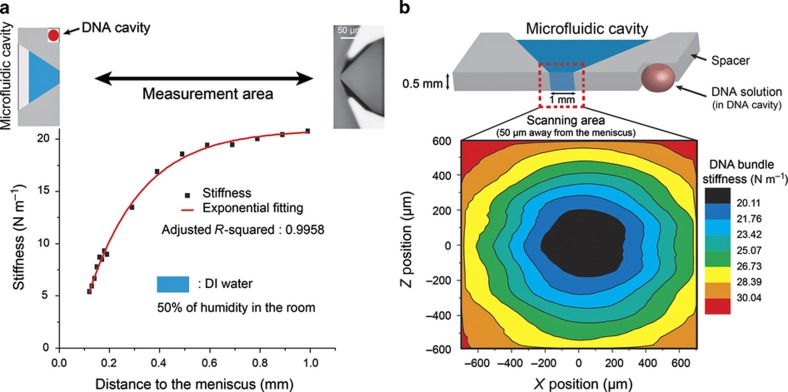
Detection of the centre of the microfluidic cavity with a trapped DNA bundle. (**a**) DNA bundle stiffness at different distances from the meniscus of the microfluidic cavity. (**b**) 2D mapping of the DNA bundle stiffness (50 μm in front of the opening of the microfluidic cavity). The stiffness is minimal at the highest humidity location, which is the middle of the microfluidic cavity opening.

DNA trapping is based on a combination of dielectrophoresis (DEP)^21^ and lateral combing as explained elsewhere^22^. A drop (3 μl) of double-stranded λ-DNA (48.5 kbp, 16 μm in length) solution diluted in deionized water is introduced into the ‘DNA cavity’ on the side of the main cavity (Figure 3). Using the nano-robot, the tips of the SNT are inserted approximately 30 μm inside the DNA solution. Then, DEP is performed by applying a potential difference (1 MV m^−1^ at 1 MHz) between the SNT tips. Due to the aluminium coating of the tips, DNA molecules attach randomly to the SNT^23,24^ and are extended by DEP^21^. Moving the nano-robot laterally at 20 μm s^−1^ (for 2 mm) allows consequent removal of the SNT tips out of the DNA solution. When the first tip is out of the solution, the attached DNA molecules are extended due to the receding air-liquid interface. For better extension performance, the lateral speed of the nano-robot is dropped to 1 μm s^−1^ until the second tip moves out of the liquid while forming the bridging DNA bundle of ~10 μm in length defined by the gap between the tips (Supplementary Figure S1). The DEP voltage is then turned off before entering the main cavity for the real-time monitoring of the DNA bundle characteristics.

### Automatic insertion of the DNA into the microfluidic cavity

Owing to the encircling glass and spacer, the meniscus has a non-uniform geometry. Insertion of the SNT at a random position can cause additional force on the mobile arm and thus interferes with the measurements. To minimize this effect, the SNT has to be inserted at the mid-point of the meniscus to obtain a symmetrical force field with the following automated procedure.

The exponential dependency between the DNA stiffness and ambient humidity (assuming a linear humidity drop with distance from the liquid source in Figure 3a) provides an accurate method of positioning the SNT. The SNT and DNA bundle are used as a high-accuracy humidity sensor for precise self-positioning relative to the meniscus. A 2D-scan of the entire cavity front (Figure 3b) at an elevated continuous speed (100 μm s^−1^) revealed a Gaussian-like map of the DNA bundle stiffness, which is related to the humidity around the cavity. To obtain the centre position of the meniscus, only two scans are necessary in the *X* and *Z* directions. The minimum of the stiffness identifies the middle of the cavity as the (*x*, *z*) coordinate with the highest humidity. At this position, the DNA bundle captured between the SNT tips is then inserted into the meniscus at a constant speed (5 μm s^−1^). At the end of the experiment, the DNA bundle is removed, and the mechanical characteristics of the bare SNT are recorded again at the exact position of the measurements. This final measurement determines the reference *F*_T_ of the SNT without DNA but including the liquid interface for the calculation of the bundle mechanical properties and its evolution during the irradiation using Equation (1).

### Evaluation of the SNT under irradiation

**Figure 4:**
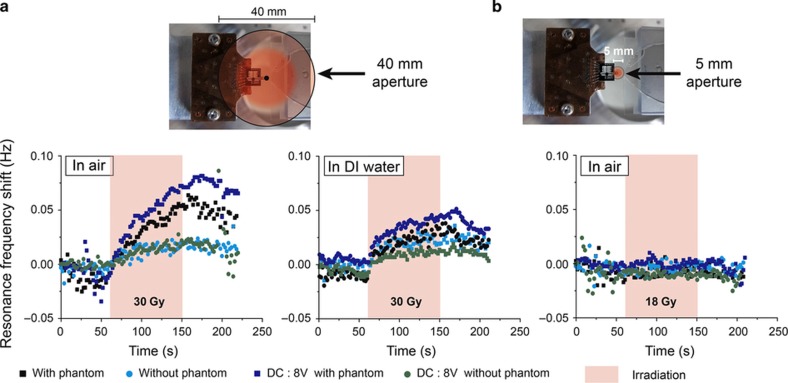
Control experiments to evaluate the influence of irradiation on the resonance frequency of a bare SNT. (**a**) The irradiation beam (40 mm aperture) is aligned with the tips of the SNT first in air and then in DI water. The resonance frequency of the bare SNT during irradiation is plotted for four different parameters. The phantom, a water equivalent material, is placed at the top of the SNT to mimic the skin of the patient. A direct current (DC) voltage of 8 V is applied on the actuator to evaluate possible leakage currents. (**b**) In air experiment with a beam aperture of 5 mm.

The X-ray beam generates an extremely harsh electromagnetic environment, which could dramatically degrade the integrity and sensing capability of the proposed system. The equipment, computer and electronic apparatus are placed at ~1 m from the beam for protection. Although the SNT's body is grounded to limit charge accumulation, the bare SNT is irradiated under various conditions to evaluate the measurement stability and induced electronic noise. The CyberKnife beam is collimated through different apertures and focused on the SNT sensor and actuator parts in air and with the tip immersed in solution. To estimate noise generation by possible leakage current, SNT actuators are also polarized with high DC voltage during irradiation. Finally, these experiments are performed with and without a phantom, a plastic shell covering the SNT and the cavity that mimics the biological layer above the targeted tissues. This surface layer guarantees the condition of electrical equilibrium in the tissues exposed to the higher irradiation dose. In the large aperture case, that is, the worst possible conditions, 30-Gy irradiation affects the capacitances by accumulation of charges and results in a resonance frequency shift of 0.1 Hz (Figure 4a). To reduce this irradiation effect on the SNT, all experiments are performed with a 5 mm aperture, for which the frequency drift is contained in the PLL noise (Figure 4b).

## Results

### Irradiation in air

**Figure 5:**
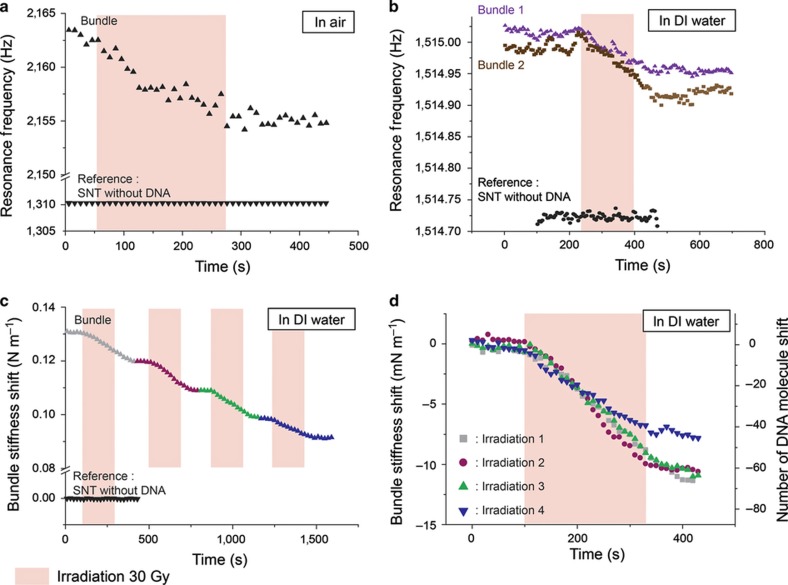
Irradiation of DNA bundles in air and deionized (DI) water. (**a**) Variation of the resonance frequency of the SNT+ DNA irradiated in air compared to the same experiment without DNA for reference. (**b**) Comparison of the irradiation effects on the resonance frequencies of two different DNA bundles in DI water trapped with the same SNT. (**c**) Shift of the DNA bundle stiffness in DI water during four successive irradiation cycles. (**d**) Comparison of the irradiation effect on the DNA bundle stiffness for the four consecutive irradiation cycles in (**c**). The right axis corresponds to the approximate number of DNA molecules damaged according to the stiffness value of a single molecule with a length equal to the gaps between SNT tips.

The plot in Figure 5a shows the real-time variation of the resonance frequency (*F*_T+DNA_) of an SNT with trapped DNA in air during 30-Gy of irradiation for 220 s. As the control reference without DNA did not show significant frequency changes under the same conditions, the decrease in the resonance frequency of the system, *F*_T+DNA_, originates from the degradation of the DNA bundle, most likely induced by the irradiation.

### Irradiation in DI water

The proposed system provides repeatable experiments owing to precise placing of the SNT relative to the cavity. For example, the same SNT and cavity are used in two separate runs (Figure 5b), in which two different DNA bundles are trapped and placed in deionized (DI) water. The initial resonance frequency shifts (in DI water) due to trapped DNA bundles are similar and are approximately 0.3 Hz higher than the reference measurement. For both bundles, the resonance frequency values are stable before and after the 30-Gy irradiation. However, during the 150-s irradiation time (red shaded regions), a smooth and significant decrease in the resonance frequency is observed. At this stage, without further confirmation by chemical analysis, it is impossible to correlate the damage of the DNA bundle with direct or indirect damages to the single molecules let alone distinguish between the accumulation of SSBs (single-strand breaks) and DSBs (double-strand breaks) or other types of molecular defects. Nevertheless, the correlation between the irradiation time and the resonance frequency decrease, signifying the degradation of the mechanical characteristics of the trapped DNA bundle, is evident. As the irradiation in DI water experiments causes direct and indirect damage to the DNA bundle, the effect should be higher than for irradiation in air (suffering from direct damage only). As a result, the decrease in the resonance frequency relative to the initial shift due to the DNA bundle is an order of magnitude larger for irradiation in DI water than in air, although the decrease is much higher in the in air experiments because a DNA bundle in air is drier and thus much stiffer than a DNA bundle in DI water (Figures 5a and b).

### Multiple irradiation cycles on the same bundle of DNA in DI water

A DNA bundle is trapped between the SNT tips and irradiated in DI water for four consecutive 210-s sessions of a 30-Gy dose with a recovery time of 180 s between the sessions (Figures 5c and d). Figure 5c shows the variation in DNA bundle stiffness throughout the experiment. For a more quantitative study, the four sets of irradiation periods are superimposed in Figure 5d by aligning both their starting time and bundle stiffness prior to each irradiation period. Figure 5d also gives an order of magnitude to the equivalent number of broken molecules during each irradiation period by scaling the bundle stiffness change by the single ds-DNA molecules^9^. The decrease of frequency (possibly corresponding to molecular damage) is highly reproducible for the first three irradiation steps, while for the fourth step, the effect of the irradiation seems to be reduced (Figure 5d). This could be interpreted as a type of ‘saturation’ effect if we suppose that the DNA bundle at this stage could already be strongly damaged. This experiment on a unique DNA bundle demonstrates the short-term repeatability of the irradiation effects, and moreover, it provides some clues about the kinetics of DNA degradation.

## Theoretical model

The experimental results described in the previous section underline the complexity of the ionizing radiation effects on the mechanical response of a DNA bundle. Moreover, it should be considered that the experimental conditions of the DNA molecules in the SNT experiments are far from the environment of a cell nucleus, where the DNA is tightly packed in the chromatin fiber coiled around the histone proteins. Therefore, a theoretical modeling of DNA degradation represents important support for scientific analysis of the experimental results in view of the quantifying protocols for clinical research objectives.

Looking at the curves in Figure 5, it is difficult to deduce a common kinetic behaviour for the degradation of the bundle stiffness; the decrease of the frequency *F*_T+DNA_ may appear to follow a roughly linear, a power-law, or possibly an exponential decrease. In our previous studies^25,26^, we demonstrated that the behaviour of the effective bundle stiffness at a low density of breaks and with vanishing DNA–DNA interactions within the bundle should be exponential in the number of breaks based on purely probabilistic arguments. However, the interactions between roughly parallel DNA fibers in the bundle cannot be neglected in a more realistic model. Indeed, the lateral interaction is mediated by the solvent and has both an electrostatic and dispersive (Van der Waals) nature. Moreover, the experimental arrangement of the DNA fibers in the bundle is poorly controlled, and it cannot be excluded that some fibers could be attached by both ends to the SNT tips, although some others have one loose end and could probably be sticking to or knotting around nearby fibers. In addition, similar knotting and sticking could occur for some DNA strands after partial or complete breaking by the radiation. In the following, we discuss a more detailed theoretical model for the breaking kinetics of the irradiated DNA bundle, allowing a direct comparison with the experimental results. We assume that in standard conditions, the irradiation generates a uniform break rate *b* (breaks per second) in the entire structure composed of *M* parallel fibers each of length *l* clamped at the ends. A living cell under irradiation experiences ~40 DSB under a dose of 1 Gy on its entire DNA composed of 3×10^9^ base-pairs with a spacing of ~3.4 Å per pair. In our experiments, the total length of DNA exposed to irradiation is *Ml*=0.03 m (the values are detailed in the explanation for Equation (7)); therefore, 1 Gy should proportionally produce 1.2 breaks. Hence, with an irradiation of 120 Gy (4×30 Gy), our value of *b* should be 120×1.2/1375=0.1 s^−1^, where *T*=1375 s is the total irradiation time. Nevertheless, this scaling of the rate would make sense only in living conditions, where the DNA is wrapped around the histones to form the chromatin fiber and is protected by cell and nuclear membranes as well as by other cellular structures. For this reason, it seems reasonable that a completely free DNA bundle openly exposed to the incoming radiation and reactive species created in the solvent by the same radiation may experience a somewhat higher break rate *b.* Consequently, a value of *b*=1 s^−1^ will be adopted in the model. In addition, a mechanism of self-healing of the DNA breaks could be considered. This mechanism may be represented by a type of ‘collision’ of some broken DNA fibers, which make new links in the bundle, including knotting and sticking via dispersion forces. Such knotting increases the effective bundle stiffness, and thus, the event can be represented as a new ‘repaired’ molecule. If we imagine two pieces A that make a new link B, this reads similar to a reaction as follows: A+A→B. The reaction rate would read as $-\frac{1}{2}.\frac{\mathrm{dA}}{dt}=\frac{\mathrm{dB}}{dt}$ (rate of disappearance of two ‘A’s equal to the rate of appearance of one ‘B’), and the rate of disappearance of ‘A’s would then be proportional to the squared concentration of A, that is $\frac{\mathrm{dA}}{dt}=-w[A][A]=-w{\left[A\right]}^{2}$, hence the second-order term in the number of breaks *N* at time *t*. The sum of DSB creation at a rate *b* plus this ‘healing’ mechanism is described with second-order chemical kinetics:

(3)dNdt=b−bβN2

The kinetic constant is rewritten as *w*=*βb*, and the nondimensional parameter *β* represents a sort of ‘strand healing coefficient’. This differential equation is able to account for a self-reparation mechanism, which possibly explains a type of saturation effect of the DNA bundle stiffness for long irradiation times.

Equation (3) is easily solved with the following solution:

(4)N=1βtanh(bβt)

The limit of this solution at very long irradiation times is

(5)limt→∞N=1β

This last equation shows that the number of breaks tends to approach a finite value, that is, the breaking mechanism with ‘reparation’ leads to a saturation of the decrease of the overall stiffness, which should approach a finite value as well.

On the other hand, in a large interval of values of *N* (such that the broken DNA fragments retain a length comparable to the SNT spacing, that is, a relatively low density of strand breaks) the degradation of the mechanical stiffness *E*_eff_ (effective Young’s modulus) can be written in this model in terms of the number of breaks, *N*, as follows:

(6)keff=EeffAl=MEAlexp[−φ(α)NM]

as demonstrated in recent theoretical studies from our research team^25,26^. Here, *φ* is a universal function depending on the parameter *α*=*k*_int_*l*^2^/*E*, including the single-molecule stiffness *E* and the viscoelastic interaction coefficient *k*_int_ between the fibers of the bundle. This equation shows that the effective stiffness of the bundle decreases exponentially with the number of DSBs. The second-order kinetics solution can be combined with the exponential degradation by replacing *N* in Equation (5) with the result of Equation (6), thus yielding the final expression:

(7)keff=MEAlexp[−φ(α)tanh(bβt)Mβ]

Here the fixed parameters are as follows: a Young’s modulus of one DNA molecule of *E*=350 MPa; a DNA average radius of *R*=1 nm, corresponding to a cross-section area of *A*=*πR*^2^~3.2 nm; an initial length of DNA strands trapped between the tweezer tip gap of *l*=15 μm per molecule; and an initial number of DNA molecules estimated at *M*=1800 from the experimental data (ratio between the stiffness of the initial bundle and those of a single ds-DNA molecule^9^). From this equation, the value of the unknown function *φ* at the argument *α*^1/2^ and the value of the parameter *β* can be determined.

For small *N*, that is, in the limit *t*→0, the slope of the stiffness is given by:

(8)dkeffdt(t→0)=-EAlφ(α)b

Therefore, this provides an estimate of the product $\varphi (\sqrt{\alpha })b=0.7$ (in units of s^−1^). Since we set *b*=1 s^−1^, *φ*=0.7 is obtained. It is worth noting that such an estimated value for the unknown function *φ* is consistent with the corresponding values derived in Ref. 21 for various other geometrical and coupling conditions.

**Figure 6:**
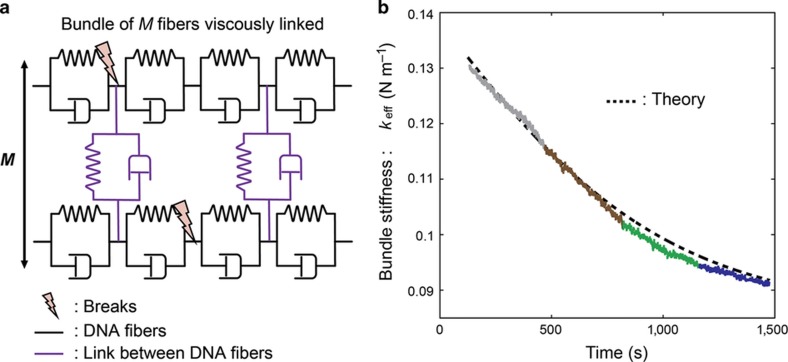
Comparison between the experiments and theoretical model. (**a**) The DNA bundle is schematized as composed by *M* molecules in parallel modelled with a series of visco-elastic dash pots. The confinement also brings a lateral coupling between the molecules, also modelled by visco-elastic components in blue. The DNA strand breaks are simulated by inserting random breaks in the visco-elastic chain (black). It should be noted that the lateral coupling allows the same molecule to support some stress also after being broken at various lengths. (**b**) Comparison of the calculated DNA bundle stiffness degradation under a constant damage rate (dashed curve) and the experimental data from Figures 5c and d (colored segments).

A comparison of the effective bundle stiffness *k*_*eff*_ from the experiments and the model is reported in Figure 6b as a function of the irradiation time. The multicolor curves are the experimental values after removing the discontinuities corresponding to the non-irradiation time windows; the black-dashed curve is the theoretical bundle stiffness behaviour predicted by Equation (7) with a fitted ‘healing’ coefficient *β*=8.1×10^−7^. It can be observed that the model with second-order kinetics provides a very good interpretation of the experimental results for both the decay time of the degradation and saturation effect with increasing damage.

## Conclusions

We used a MEMS-based device, the SNT, to perform the first real-time detection of ionizing radiation damage to DNA by means of mechanical characterizations of a bundle of DNA molecules irradiated both in air and in liquid. A direct correlation between the mechanical degradation of DNA bundles and the radiation dose of the gamma-ray beam was demonstrated. Control experiments were performed to rule out other possible causes of the observed variations of the frequency and amplitude of the mechanical oscillation of the SNT. The good repeatability of the experiments and a correlated theoretical analysis allowed the mechanics of real-time DNA damage under irradiation to be studied. However, although the correlation between degradation and radiation is very consistent, at this stage it is not yet possible to directly attribute the mechanical degradation to a molecular scale sequence of events, such as specific types of DNA breaks.

By considering the low cost, and ease of fabrication, characterization, and manipulation of the MEMS devices and the associated microfluidic set-up, such results pave the way for further studies aimed at optimizing tumor treatment using ionizing radiation. More clinically relevant research objectives could be addressed in the immediate future by methods such as immersing the DNA bundles in a solution containing various radiation sensitive molecules, reactive oxygen species, proteins, and enzymes from cell nuclear extracts, with the aim of defining a patient-specific radiation treatment for future personalized medicine protocols.
