# Supplementary material for: Real-time mechanical characterization of DNA degradation under therapeutic X-rays and its theoretical modeling
Source: Microsyst Nanoeng. 2016 Dec 5;2:16062. doi: 10.1038/micronano.2016.62 (PMC6444744; doi:10.1038/micronano.2016.62)
Supplement: Supplementary Information [file micronano201662-s1.pdf]

Supplementary file

Real-time mechanical characterization of DNA degradation under therapeutic X-rays and its theoretical modeling

Grégoire Perret[1,2,3], Thomas Lacornerie[4], Fabio Manca[2], Stefano Giordano[2], Momoko Kumemura[1,3,5], Nicolas Lafitte[1], Laurent Jalabert[1], Mehmet C. Tarhan[1,3,5], Eric F. Lartigau[3,4], Fabrizio Cleri[2,3], Hiroyuki Fujita[1,3,5] and Dominique Collard[1,3,5]

Microsystems & Nanoengineering (2016) **2,** 16062; doi:10.1038/micronano.2016.62; Published online: 5 December 2016

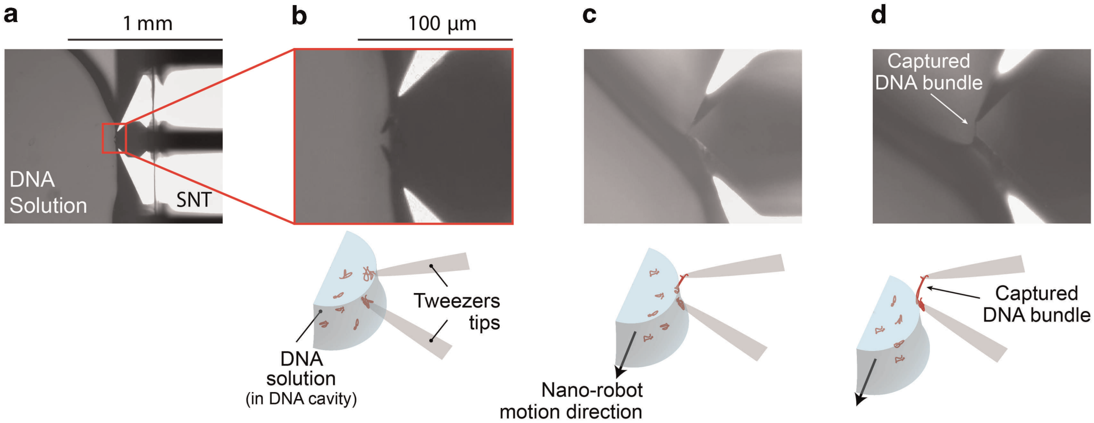

Figure S1 DNA trapping by the SNT (**a**) Using the nano-robot, the tips of the SNT are inserted approximately 30 µm in a drop (3 µl) of double-stranded λ-DNA (48.5 kbp, 16 µm in length) solution diluted in deionized water. (**b**) DEP is performed by applying a potential difference (1 MV m – 1 at 1 MHz) between the SNT tips. (**c**) Moving the nano-robot laterally at 20 µm s^{-1} (for 2 mm) allows consequent removal of the SNT tips out of the DNA solution. (**d**) When the first tip is out of the solution, the attached DNA molecules are extended due to the receding air-liquid interface.

[1]LIMMS/CNRS-IIS UMI 2820, Institute of Industrial Science, The University of Tokyo, 4-6-1 Komaba Meguro Ku, Tokyo 153-8505, Japan; [2]IEMN, UMR8520, CNRS, Avenue Poincaré Cité Scientifique, BP 60069, Villeneuve d'Ascq, Cedex 59652, France; [3]CNRS/IIS/COL/Lille 1 SMMiL-E project, CNRS Délégation Nord-Pas de Calais et Picardie, 2 rue de Canonniers, Lille, Cedex 59046, France; [4]Centre Oscar Lambret, Université de Lille, Département Universitaire de Radiothérapie, Centre Oscar Lambret, Lille 59000, France and [5]Institute of Industrial Science, The University of Tokyo, 4-6-1 Komaba, Meguro-ku, Tokyo 153-8505, Japan.
Correspondence: Grégoire Perret (gperret@iis.u-tokyo.ac.jp) or Dominique Collard (collard@iis.u-tokyo.ac.jp)
